# Management of patients with extensive locally advanced thyroid cancer: results of multimodal treatments

**DOI:** 10.1007/s40618-023-02234-w

**Published:** 2023-11-30

**Authors:** A. Prete, E. Pieroni, E. Marrama, L. Bruschini, M. Ferrari, G. Scioti, V. Aprile, F. Guarracino, C. E. Ambrosini, E. Molinaro, R. Elisei, M. Lucchi, G. Materazzi

**Affiliations:** 1 Endocrine Unit, Department of Clinical and Experimental Medicine, University Hospital of Pisa, Via Paradisa 2, 56124 Pisa, Italy; 2 Endocrine Surgery Unit, Department of Surgical, Medical and Molecular Pathology and Critical Area, University Hospital of Pisa, Pisa, Italy; 3 Thoracic Surgery Unit, Department of Surgical, Medical and Molecular Pathology and Critical Area, University Hospital of Pisa, Pisa, Italy; 4 Ear Nose Throat (ENT) Audiology and Phoniatric Unit, Department of Surgical Pathology, Medical, Molecular and Critical Area, University Hospital of Pisa, Pisa, Italy; 5 Vascular Surgery Unit, Department of Translational Research and New Technologies in Medicine and Surgery, University Hospital of Pisa, Pisa, Italy; 6 Section of Cardiac Surgery, University Hospital of Pisa, Pisa, Italy; 7 Cardiothoracic and Vascular Anaesthesia and Intensive Care, Department of Anaesthesia and Critical Care Medicine, University Hospital of Pisa, Pisa, Italy

**Keywords:** Advanced thyroid cancer, Multidisciplinary team, Tyrosine kinase inhibitors

## Abstract

**Purpose:**

Surgery plays a key role in the treatment of thyroid cancer (TC) patients. Locally advanced cases, however, can require an extensive surgical approach with technical issues and a high risk of complications. In these cases, a multidisciplinary evaluation should be carried out to evaluate pros and cons. The aim of this study was to share our experience, as a multidisciplinary team, in the management of patients with locally advanced TC with a particularly extensive local disease, whose surgical approach could be challenging and part of a multimodal treatment.

**Methods:**

We retrospectively evaluated clinical, surgical, and oncologic features of all patients with locally advanced TC who had undergone multidisciplinary surgery from January 2019 to June 2020.

**Results:**

Six patients (two cases each of poorly differentiated, papillary, and medullary TC) were included. Four out of six were suffering from symptoms related to the advanced disease. At pre-surgical evaluation, a multidisciplinary team proposed extended surgery with radical intent via cervicotomy and sternotomy, considering other therapies not feasible or probably ineffective without it. No one passed away in intra- or perioperative time. At the end of follow-up (median 2.6 years), all patients presented a remission of symptoms due to the advanced disease, four patients were submitted to adjuvant therapies and only one patient died for a cause unrelated to the disease.

**Conclusion:**

This series of very advanced TCs shows the effectiveness of a surgery performed by a multidisciplinary team in controlling symptoms, allowing adjuvant therapies, and improving the survival of patients whose cases would otherwise be very difficult to manage.

## Introduction

Thyroid cancer (TC) is the most frequent endocrine tumor, accounting for about 22 new cases per 100.000 people every year [1]. Malignant transformation can happen both in follicular and parafollicular thyroid cells. Differentiated thyroid cancer (DTC), that represents 90% of thyroid malignancies and includes papillary thyroid cancer (PTC) and follicular thyroid cancer (FTC), poorly differentiated thyroid cancer (PDTC), and anaplastic thyroid cancer (ATC), originates from follicular cells, while medullary thyroid cancer (MTC) originates from parafollicular cells [2, 3].

Although an excellent prognosis has been widely recognized for thyroid malignancies [4], metastatic DTC, as well as specific hystotypes, namely, ATC, PDTC, or MTC, have worse prognosis [4, 5]. Durante et al. reported an overall survival of 42% at 10 years in patients with metastatic DTC at diagnosis, which dropped to 10% in radiorefractory cases [6]. In PDTC, the 5 year overall survival was recently reported to range from 63.6 to 72.8% [7, 8] and only one-third of patients reached clinical remission during their clinical course [8]. A slightly better clinical outcome was recently shown by Matrone et al. [9] in patients with MTC, although almost 50% of patients did not experience an excellent response during a median follow-up of 7 years.

Surgery plays a key role in the therapeutic management of patients with TC even in locally advanced or metastatic stage [4, 10]. In these latter cases, especially when the tumor is not confined to the neck, the role of surgical resection is still being discussed [11]. Moreover, the definition of locally advanced TC, albeit intuitive, is not standardized, as well as the definition of “unresectable” cases [11]. Finally, an extensive surgical treatment could be controversial both for technical issues and the risk of complications in patients who often present with multiple comorbidities [11, 12]. In these cases, systemic therapies could be considered and represent the standard of care [13].

The aim of this study was to share our experience, as a multidisciplinary team, in the management of patients with locally advanced TC with a particularly extensive local disease whose surgical treatment could be challenging and not resolving but at the same time necessary to make other medical treatments possible.

## Materials and methods

We retrospectively reviewed patients with locally advanced TC, in whom the surgical approach of the disease needed the presence of different type of surgeons (i.e. vascular, cardiovascular, endocrine, and thoracic surgeon) because of the local aggressiveness. All patients were preoperatively evaluated by a multidisciplinary team comprising of an endocrinologist, an anesthesiologist, and surgeons of these different specialties from January 2019 to June 2020. The cases included in the present study were only those in whom surgical treatment (although extensive) was considered feasible after multidisciplinary evaluation. Conversely, we excluded all the advanced cases in whom surgical treatment was not feasible according to the judgment of different surgeons.

We analyzed demographic, clinical, surgical, and pathological data of all patients using our medical records. All patients included in this study had a complete preoperative work-up. In this regard, they were evaluated with a total body CT-scan, neck ultrasound and, when necessary, gastroscopy, bronchoscopy, and cavography. As for policy of our hospital, all patients signed an informed consent for the use of their clinical and biochemical data for research purposes. They also signed an informed consent to proceed with the suggested interventional treatments. The present study has been approved by the local ethical committee.

## Results

Six patients with the above-described clinical features were identified. From a histological point of view, they were 2 PDTCs, 2 PTCs, and 2 MTCs. Four patients underwent major surgery, performed by a multidisciplinary team, including a thoracic and an endocrine surgeon (patients #3–6). In the other two cases (patients #1 and #2), vascular and cardiac surgery expertise was also necessary because of neoplastic vascular and cardiac involvement requiring the use of extracorporeal circulation (ECC), cardioplegia, and vascular graft placement.

### Presurgical evaluation

Extensive surgery was planned in all 6 patients: in 3 patients (#1, #2 and #3) as initial treatment and in the other 3 (#4, #5 and #6) after a previous initial treatment (total or near-total thyroidectomy). In particular, patient #4 presented a MTC (T1aN1aM0, stage III according to AJCC 8th edition), patient #5 a PDTC (T4NxM1, stage IV), and patient #6 a PTC (T2NxMx, stage I). In patients #3, #4, and #5, preoperative diagnosis was performed by fine-needle aspiration (FNA), while in two cases (#1 and #6), it was obtained by tru-cut [14] and in one case (#2) by ultrasound transbronchial needle aspiration (EBUS-TBNA).

Table 1 shows disease extension for each patient. Mediastinal lymph nodes were the most common site of disease (5 out of 6 patients), although laterocervical ones were also commonly involved (4 out of 6 patients). Interestingly, all patients experienced at least two sites of disease. Patient #5 also presented with distant metastases (lung).

**Table 1:** Neoplastic thyroid disease localization and surgical procedure in each patient

	Pre-surgical diagnosis	Thyroid	Latero-cervical lymph nodes (dimension of the biggest)	Mediastinal lymph nodes (dimension of the biggest)	Vascular infiltration	Distant metastasis	Surgical procedure
Patient 1	PDTC	Yes			Yes (right jugular vein and superior vena cava)		Total thyroidectomy, cervical lymphadenectomy and cavo-atrial thrombectomy
Patient 2	PTC	Yes		Yes (6 cm)	Yes (anonymous artery and left brachiocephalic vein)		Total thyroidectomy, mediastinal debulking
Patient 3	MTC	Yes	Yes (5 cm)	Yes (3 cm)			Total thyroidectomy and cervical and superior mediastinal lymphadenectomy,
Patient 4	MTC		Yes (2.0 cm)	Yes (0.8)			Cervical and superior mediastinal lymphadenectomy
Patient 5	PDTC		Yes (4.0 cm)	Yes (3.2 cm)		Yes (centimetric lung metastasis)	Central cervical and superior mediastinal compartment lymphadenectomy
Patient 6	PTC	Yes (Local recurrence)	Yes (1.2 cm)	Yes (2 cm)			Central and left laterocervical and superior mediastinal compartment lymphadenectomy
Total (%)	–	4 (66.7%)	4 (66.7%)	5 (83.3%)	2 (33.3%)	1 (16.7%)	–

A multidisciplinary evaluation was performed on all patients. In patients #1 and #2, radiotherapy and multikinase inhibitors’ treatments were not feasible due to a very high embolic risk; at the same time, their vascular invasion was considered life-threatening. In the other cases, surgery was indicated as the first therapeutic approach to achieve local disease control.

In their clinical history, all patients presented with co-morbidities: four patients experienced major cardiovascular disease (ischemic heart disease, heart failure, stroke and abdominal aorta aneurysm, and celiac tripod aneurysm), two were suffering from obesity with arterial hypertension, and one had undergone hysterectomy for uterine fibromatosis (Table 2). At the moment of surgery, all these comorbidities were considered clinically compensated thanks to their pharmacological treatment.

**Table 2:** Patients’ comorbidities and epidemiological data

	Age at surgery (years)	Sex	Major cardiovascular disease	Arterial hypertension	BMI (Kg/m^2^)	Diabetes	Dyslipidemia	Other comorbidities
Patient 1	69	Male	Yes (stroke)	Yes	26.7	Yes	Yes	
Patient 2	75	Female	Yes (heart failure)	Yes	26.8		Yes	
Patient 3	47	Female			17.8			Yes (uterine fibromatosis)
Patient 4	66	Male	Yes (abdominal aorta aneurysm and celiac tripod aneurysm)	Yes	25.2		Yes	
Patient 5	68	Male	Yes (ischemic heart disease)	Yes	35.6	Yes		
Patient 6	65	Female		Yes	31.6			Yes (non-functional adrenal adenoma)
Total (*n* = 6)	Median 65.5	3:3	4 (66.6%)	5 (83.3%)	2 (33.3%) with BMI ≥ 30 kg**/**m^2^	2 (33.3%)	3 (50.0%)	2 (33.3%)

**Fig. 1:**
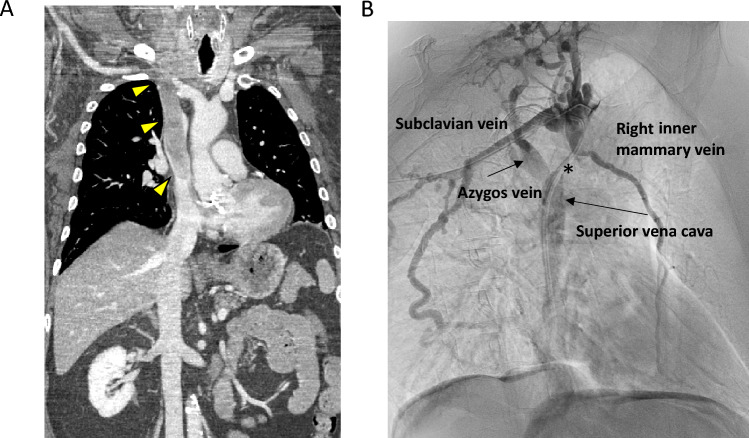
**A** Extensive thrombus from the right jugular vein to the right atrium (yellow triangles) of patient #1 at CT-scan. **B** Superior vena cava obstruction due to the presence of thyroid metastasis (asterisk) of patient #2 at superior vein cavography

Moreover, at admission, 4 out of 6 patients suffered from symptoms related to their advanced disease. Patients #1 and #2 presented with mediastinal syndrome due to an extensive thrombus from the right jugular vein to the right atrium (Fig. 1A), and to an obstruction of the superior vena cava (Fig. 1B), respectively. In particular, they presented edema of neck, arms, and trunk, distended cervical and superficial chest collateral veins and cough. Patient #3, who was affected by MTC, presented with chronic secretory-type diarrhea, due to high levels of serum calcitonin, while patient #4 referred a severe and long-standing pain in the left laterocervical site.

### Surgical treatments

At the time of surgery, the median age was 67.5 years (IQR 65.5–68.5, range 47–75). In all patients, surgery was performed through cervicotomy in order to remove any possible local recurrence and ensure an adequate bilateral neck dissection, and then through complete median sternotomy to isolate all mediastinal structures from the lymphadenopathy.

**Fig. 2:**
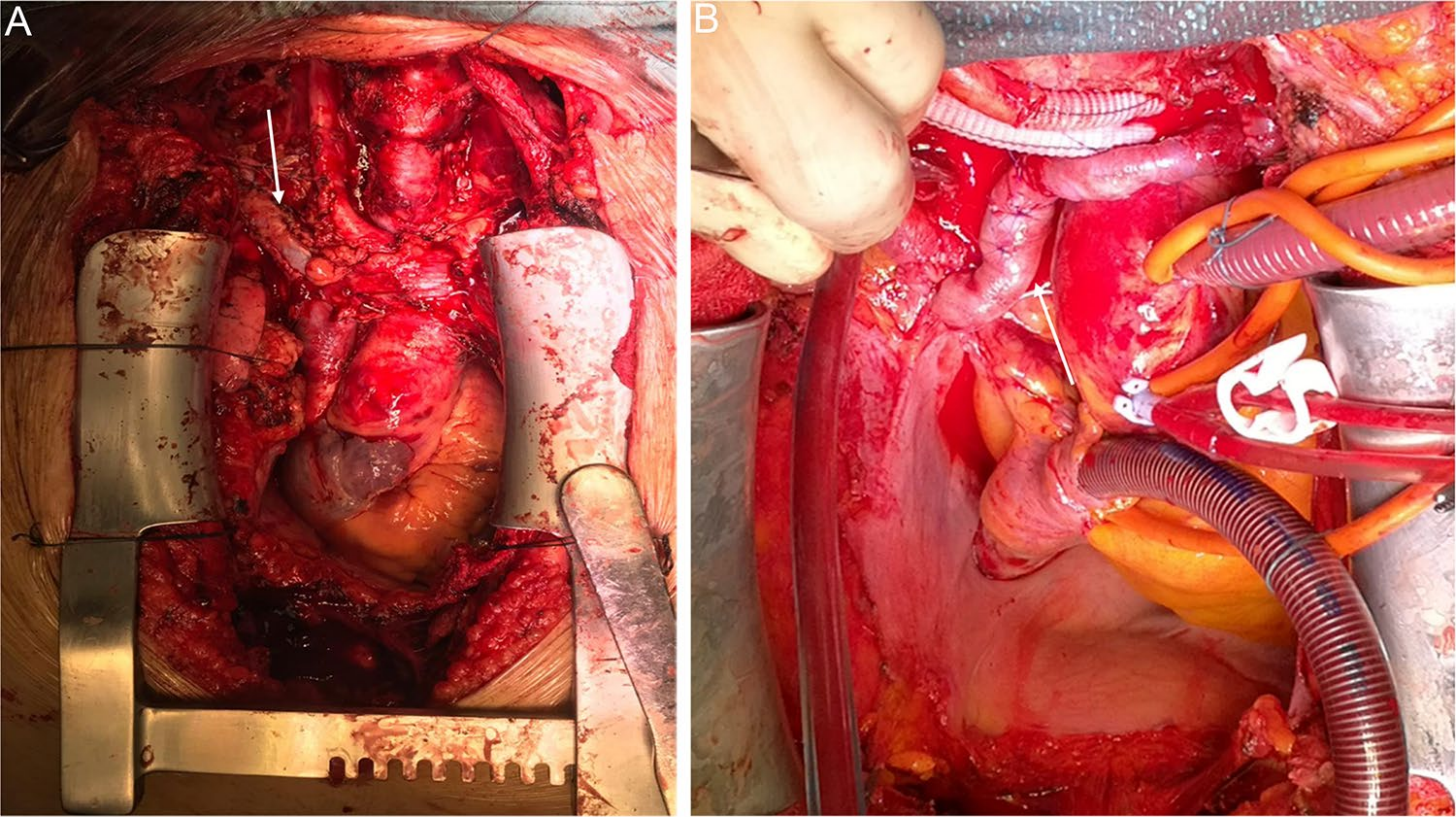
**A** Superior vena cava reconstruction (white arrow) with bovine pericardial patch of patient #1. **B** Left innominate vein reconstruction (white arrow) with Dacron grafts of patient #2

In patient #1, a radical thyroidectomy by peeling the tracheal surface and an extended cervical lymphadenectomy en-bloc with external right jugular vein and superior vena cava were performed, combined with cavo-atrial thromboarterectomy and vascular reconstruction with bovine pericardial patch (Fig. 2A). Likewise, patient #2 had a neoplastic infiltration of the anonymous artery and of the left brachiocephalic vein requiring vascular resection and reconstruction with Dacron grafts, and an endoluminal thrombus of the superior vena cava that was removed (Fig. 2B).

In 5 cases, we obtained a macroscopic complete resection confirmed by the histopathological examination (R0). In one case (patient #1), a macroscopic complete resection was not possible because of tracheal infiltration requiring surgical debulking by tracheal shaving (R2).

Wide lymph-node resection was performed in patient #2–6. In particular, 1 metastatic mediastinal lymph node (6 cm) with extranodal extension and pleural infiltration was surgically removed in patient #2, multiple and confluent cervical (biggest one of 5 cm) and mediastinal metastatic lymph node with wide extra nodal extension (biggest one of 3 cm) in patient #3, 24 metastatic cervical out of 50 surgically removed (biggest one of 2.0 cm) and 10 metastatic mediastinal out of 21 surgically removed (biggest one of 0.8 cm) lymph nodes in patient #4, 1 metastatic cervical of 4.0 cm out of 2 surgically removed and 1 mediastinal lymph node of 3.2 cm out of 3 surgically removed in patient #5, and 3 metastatic cervical out of 11 surgically removed (biggest one of 1.2 cm) and 1 of mediastinal lymph node of 2 cm out of 4 surgically removed in patient #6. Interestingly, in patients #4, #5, and #6, who have been already submitted to a previous surgery treatment, we observed a histological concordance between lesions surgically removed during initial and current treatment.

### Adverse events

Surgical complications were observed in 4 out of 6 patients (Table 3). All 4 patients experienced typical thyroid surgery complications (i.e., hypoparathyroidism and laryngeal nerve injury with vocal cord paralysis). In particular, patients #3 and #6 had only hypoparathyroidism, patient #2 only right laryngeal nerve injury with vocal cord paralysis, and patient #1 both. As far as atypical thyroid surgery complications were concerned, pulmonary embolism was observed in patients #1 and #6.

**Table 3:** Surgical complications grouped into typical and atypical thyroid surgery complications

	Typical thyroid surgery complications		Atypical thyroid surgery complications			
	Hypo-parathyroisism	Laryngeal nerve injury with vocal cord paralysis	Pulmonary embolism	Pneumoniae	Haemorrhagia	Sternotomy dehiscence
Patient 1	Yes	Yes (monolateral)	Yes			Yes
Patient 2		Yes (monolateral)		Yes	Yes	
Patient 3	Yes					
Patient 4						
Patient 5						
Patient 6	Yes		Yes			
Total (*n* = 6)	3 (50.0%)	2 (33.3%)	2 (33.3%)	1 (16.7%)	1 (16.7%)	1 (16.7%)

Moreover, while patients #4 and #5 did not show any clinically significant complications, patients #1 and #2 developed multiple adverse events. In particular, patient #1 developed not only an asymptomatic pulmonary embolism revealed during radiological follow-up, but also a sternotomy dehiscence that required VAC-therapy and then a second surgery. Patient #2 had postoperative bleeding requiring gauze packing and a second surgery on the second postoperative day. During intensive care unit stay, the patient developed bilateral pneumonia with pleural effusion and respiratory failure requiring transitory tracheostomy.

No complications related to the vascular graft (e.g., dislocation) nor to the ECC (i.e., nervous system complications, such as bleeding, ischemia, and multiorgan failure, in particular renal failure) were observed. In this series, no intraoperative or perioperative mortality (30- and 90 day mortality, respectively) occurred.

### Long-term follow-up

At study data lock (December 2022), patients #1–#4 did not develop the recurrence of symptoms observed before surgery. Patients #4 and #6 showed a biochemical persistence of the disease, and patients #1, #2, #3, and #5 showed a structural persistence of the disease. At data lock, 5 out of 6 patients were still alive after a median follow-up of 2.6 years (IQR 2.5–2.8, interval 2.3–3.6). Patient #1 died for reasons unrelated to the neoplastic disease after 2.5 years of good health conditions.

**Fig. 3:**
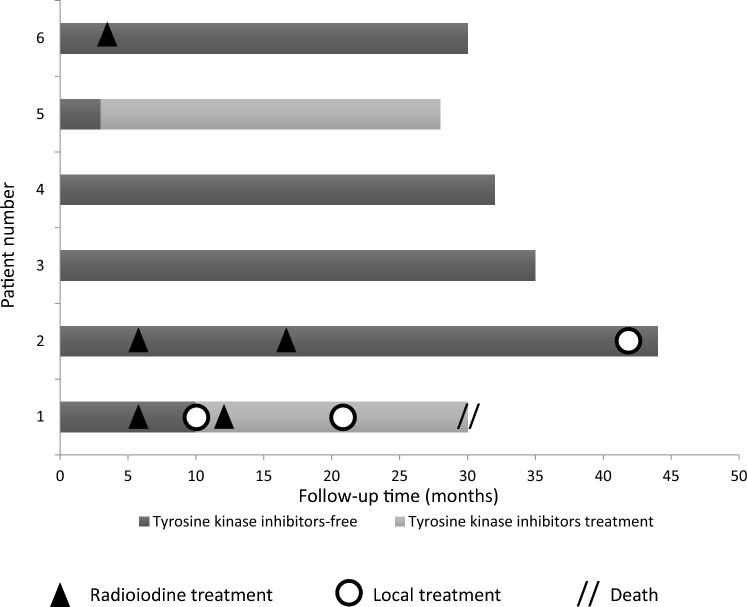
Each bar corresponds to a patient follow-up (from #1 to #6). Light and dark gray bar indicates interval with and without systemic therapies with multikinase inhibitors, respectively. Black triangle and white circle represent radioiodine treatment and local therapy, respectively

Figure 3 describes the therapies administered during follow-up. Patients #3 and #4 did not receive any further treatment after surgery. Radioiodine treatment was performed in 3 out of 6 patients (patients #1, #2 and #6), 4–6 months after surgery. Systemic therapy with multikinase inhibitor (MKI) (i.e. lenvatinib) was administered to 2 patients. Patient #1 started lenvatinib treatment 10 months after surgery for a tracheal recurrence, and also treated by endotracheal laser (twice, 10 months and 21 months after surgery), reaching local disease stabilization. Patient #5 started lenvatinib treatment for lung and a large mediastinal lymph-node metastasis that recurred 3 months after surgery, obtaining a partial response. In patient #2, a local treatment for tracheal recurrence was performed by placing an endotracheal stent.

## Discussion

In the last 20 years, the incidence of TC has progressively increased, especially since TCs with a diameter less than 1 cm are more frequently diagnosed [1]. However, many authors observed that diagnosis of TCs with a larger diameter (> 4 cm) presented a significant increase too, as well as cases with extrathyroidal extension and lymph-node metastases [15, 16]. If the challenge is to avoid overtreatment in very low aggressive cases [17], in patients suffering from advanced TC, the challenge is to be ideally radical, weighting a potential significant morbidity [11].

We reported our experience in advanced TC cases treated with a multimodal approach, including surgical, radiometabolic, and systemic therapies. We confirmed the crucial role of surgery in treating advanced TC, beyond its well-systemized role in cases confined to the neck region [18]. In our clinical series, surgery had a fundamental role in: (1) reaching a prompt symptoms resolution related to the metastatic disease, (2) allowing adjuvant therapies, not feasible or probably ineffective without a radical surgery, and (3) improving survival, as a component of a multimodal treatment regimen. On the other hand, in our series, both typical and atypical thyroid surgical complications occurred.

At admission, most of our patients presented symptoms related to the metastatic disease and, in particular, two of them mediastinal syndrome. As recently reviewed, mediastinal syndrome is very rare in TC, with a very poor prognosis [19]. Since 1879 onwards, about 60 cases of TCs with major vascular infiltration have been reported, and mediastinal vascular infiltration has been described in less than 30 cases [19–21]. In our series, surgery allowed a prompt symptoms resolution related to vascular involvement and, during a median follow-up of 2.6 years, none of our patients developed symptom recurrence, confirming that surgery should always be evaluated in case of vascular infiltration or symptomatic patients [19]. Likewise, a massive tumor debulking should be taken into consideration in locally advanced MTC patients suffering from chronic diarrhea to reduce the complaints [22], albeit new target therapies have been demonstrated to be effective, too [23]. In our case, diarrhea disappeared immediately after surgery, without recurrence during a 3 year follow-up.

Beyond symptomatic relief, local disease control is one of the main oncologic targets of an enlarged surgery [11]. In a recent report of series including 153 DTCs with invasion of subcutaneous soft tissues, recurrent laryngeal nerve, larynx, trachea or esophagus, 5 year disease-specific survival, as well as locoregional recurrence did not differ between cases with complete resection (R0) and with positive margin of resection (R1) [24]. Furthermore, regional eradication or debulking of the tumor mass, resulting in macroscopically radical surgery or in minimal residual disease, can allow other therapeutic options. In particular, two of our patients after surgery could ultimately initiate treatments with MKIs that were not feasible before surgery because of the extensive venous thrombosis. Lenvatinib, cabozantinib, and other MKIs are strong inhibitors of vascular endothelial growth factor receptor (VEGFR) and platelet-derived growth factor receptor (PDGFR) [25, 26]. Since VEGFR regulates endothelial function, it is not surprising that its inhibition predisposes to platelet activation and thrombosis [27, 28]. Bai et al*.* carried out an extensive meta-analysis about the risk of thromboembolic events during MKIs treatment in advanced TC, and observed an increased risk of arterial thromboembolic events [29]. In SELECT trial, a phase III trial evaluating progression-free survival (PFS), response rate, overall survival (OS), and toxicity of lenvatinib [30], venous thromboembolic events were observed in 5.4% of cases in the lenvatinib arm, and in 3.8% of cases in the placebo arm. Likewise, in EXAM trial, a phase III trial evaluating PFS, OS, objective response rate, and safety of cabozantinib, pulmonary embolism was observed in 2.3% of patients treated with cabozantinib, and in none of the patients in the placebo arm [31]. In line with this evidence, the multidisciplinary team decided not to start MKI in the case of such big thrombosis, while it was possible after its debulking.

Regional disease eradication or debulking could reinforce the effect of following systemic therapies, whose efficacy depends on tumor burden. An updated analysis of SELECT trial by Gianoukakis et al. showed different duration of response induced by lenvatinib according to the baseline diseases burden [32]. Radioiodine efficacy too has been related to tumor burden [33]. Song et al. showed that both biochemical and structural responses achieved after radioiodine treatment were poorer in patients with higher disease burden [34]. In our clinical series, some patients performed effective radioiodine treatments after surgery that was not decisive, but at least stabilized the disease that, in our opinion, is anyway a good clinical result in this type of patients.

In our series, despite their advanced stage and multiple comorbidities, we did not observe any perioperative deaths or any cancer-related deaths during a median follow-up of 2.6 years. Our results are in line with those of other reported series. Marcy et al. described 9 cases of TC characterized by neoplastic venous obstruction, drawn from a database of 1171 patients [35]. They argued that very invasive multimodal treatment including surgery should be performed to improve survival [35]. The same conclusion was reached by Hyer et al., who studied a series of 5 patients with venous obstruction [36]. However, this extensive surgery is not devoid of complications, both typical and atypical for endocrine surgery. About the former, 4 patients developed hypoparathyroidism and/or laryngeal nerve injury with vocal cord paralysis. However, it is well known that the risk of typical complications for endocrine surgery, such as hypoparathyroidism, is influenced by the extent of neck dissection [37, 38]. About the latter, 2 patients developed pulmonary embolism and 1 patient sternotomy dehiscence. Even though the incidence of deep venous thrombosis and pulmonary embolism is low after conventional thyroid surgery [39], in patients with diffuse neoplastic thrombosis or undergoing aggressive thoracic lymphoadenectomy the incidence is not negligible [12, 40]. All these information and evidences must be well known by the multidisciplinary team that, for these reasons, should include an expert endocrinologist in advanced TC, an endocrine surgeon with a great experience of TC and other highly experienced specialists, such as thoracic, vascular, and otolaryngologist surgeons, tracheobroncoscopist, anesthetists, and others specialists according to the extension of the disease and comorbidities of the patient.

## Conclusions

Our experience in the treatment of very advanced TCs, particularly at local level, shows the effectiveness of an extensive surgery performed by a multidisciplinary team in controlling symptoms, allowing adjuvant therapies, and improving the survival of patients whose cases would otherwise be very difficult to manage. The planning of a well-defined therapeutic strategy by a multidisciplinary team and the simultaneous presence at the operating table of expert surgeons of different disciplines determined a favorable outcome. Still relatively young patients can gain significant benefits from this therapeutic approach, mainly a longer survival and a chronicity of the disease with whom they cohabit for a long time. However, this type of surgery is possible, and should be performed, only in expert tertiary referral centers for the management of TC.
